# The role of national population-based registries in pancreatic cancer surgery research

**DOI:** 10.1097/JS9.0000000000001405

**Published:** 2024-04-04

**Authors:** Eduard Jonas, Christo Kloppers

**Affiliations:** Department of Surgery, University of Cape Town Faculty of Health Sciences, Surgical Gastroenterology Unit, Groote Schuur Hospital, Cape Town, South Africa

**Keywords:** database, national, pancreatic cancer, population-based, registry, surgery

## Abstract

Research and innovation are critical for advancing the multidisciplinary management of pancreatic cancer. Registry-based studies (RBSs) are a complement to randomized clinical trials (RCTs). Compared with RCTs, RBSs offer cost-effectiveness, larger sample sizes, and representation of real-world clinical practice. National population-based registries (NPBRs) aim to cover the entire national population, and studies based on NPBRs are, compared to non-NPBRs, less prone to selection bias. The last decade has witnessed a dramatic increase in NPBRs in pancreatic cancer surgery, which has undoubtedly added invaluable knowledge to the body of evidence on pancreatic cancer management. However, several methodological shortcomings may compromise the quality of registry-based studies. These include a lack of control over data collection and a lack of reporting on the quality of the source registry or database in terms of validation of coverage and data completeness and accuracy. Furthermore, there is a significant risk of double publication from the most commonly used registries as well as the inclusion of historical data that is not relevant or representative of research questions addressing current practices.

## Introduction

Highlights

- Registry-based studies are a complement to randomized controlled clinical trials.
- Studies based on national population-based registries have several advantages, for example, being less prone to selection bias.
- A steep increase was seen in pancreatic cancer surgery studies based on national population-based registries over the last decade.
- A minority of papers sourced data from dedicated pancreas cancer registries.
- Study format-specific guidelines were used in only 20% of publications.
- Reporting on validation of the database(s) used for sourcing data was sub-optimal, in particular, in terms of completeness and accuracy of data.

Pancreatic surgery is a central component of the multidisciplinary management of pancreatic malignancies. Pancreatic surgery may be challenging due to complicated anatomy, proximity to vital structures, and advanced disease stage, and is associated with potential serious postoperative complications, resulting in mortality and morbidity^1^. Research that creates high-level evidence through well-designed clinical studies and trials is invaluable for advancing the multidisciplinary management of pancreatic malignancies. Randomized clinical trials (RCTs) are still regarded as the most powerful clinical research tool, generating the highest levels of evidence that often result in strong treatment recommendations^2^. Registry-based studies (RBSs) play an important role in addressing research questions that are not optimally addressed in other research formats^3^. These studies usually require fewer financial resources. The intent to cover all individuals in a national population in national population-based registries (NPBRs) makes selection bias in studies based on national data less likely than in research originating from non-NPBRs registries. The purpose of this article is to provide a critical review of the potential advantages of NPBRs in pancreatic cancer surgery research while also highlighting potential shortcomings that may compromise the quality of these studies.

### Healthcare registries and registry-based research

#### Healthcare registries

Healthcare registries generate valuable data that can be used for monitoring health trends and outcomes, including epidemiology and surveillance, monitoring of treatment efficacy, and detecting adverse events associated with specific treatments or interventions. Surgical registry data are included in purely procedure-based registries or included as surgical modules in more comprehensive disease-based registries. Registry data typically include information on demographic characteristics (e.g. age, sex, and ethnicity) and health-related data (e.g. diagnoses, treatments, and treatment outcomes). The extent of populations included in registries varies from single-institutional patient cohorts to national populations.

National population-based healthcare registries intend to cover entire national populations and have several advantages compared to non-NPBRs. They allow for the calculation of incidence and prevalence at a national level, with the total country population as the denominator. Even in NPBRs with relatively low coverage, the intent to cover all individuals in a national population makes selection bias less likely than in non-NPBRs. In the Nordic countries, the creation and maintenance of nationwide population-based registries are facilitated by tax-funded healthcare systems, reporting is mandatory, and data quality is high in terms of completeness^4^. In countries with multiple healthcare sectors, including private healthcare providers, compliance is difficult to enforce, which affects data completeness^5^. The utility of registries is further enhanced if standardized personal identifiers such as social security or identification numbers are consistently used in registries. This would allow cross-referencing of data between different healthcare registries (e.g. outpatient and prescription registries), birth and total population registries, cause of death registries, and even registries containing socioeconomic data.

Assessing the validity of healthcare registries in terms of coverage, completeness, and accuracy is essential for establishing data quality. Data coverage refers to the extent to which eligible patients from the defined target population are recorded in the registry. This is usually assessed by comparing the actual registry data with other data sources, such as hospital records or cross-referencing with administrative or healthcare databases or registries^6^. Data completeness indicates the extent of missing data through simple audits of unpopulated data fields. Accuracy (correctness) is verified by comparing registry records to primary source data, such as individual patient records.

The cost of designing, implementing, and maintaining registries is a universal challenge. In many countries, the initial establishment of registries was physician-driven, often with minimal resources^7^. This is unfortunately still the case, in particular low- and middle-income countries. In high-income countries with well-financed health systems, funding for registries is more readily available. For example, in the Scandinavian countries (Sweden, Denmark, Finland, Norway, and Iceland), national registries for numerous medical conditions are fully or partially funded by central or regional authorities^4^. In these countries, registries have, over time, developed into patient-centered systems that include data on disease patterns, treatments, and treatment results linked to specific diagnoses^7^. In Sweden, this data has facilitated the creation of evidence-based national disease-specific guidelines for the management of several diseases^8^.

#### Registry-based studies and trial designs for registry-based studies

Registry-based studies have a definite and increasing role in addressing research questions that are not optimally assessable in other research formats^3^. RCTs are regarded as generating the highest levels of evidence that result in strong treatment or intervention recommendations but account for only 3–7% of publications in surgical journals^2^. However, these trials are complex, expensive, and unsuitable for addressing all research questions^2^. A major concern of RCTs is the risk of bias, in particular selection and reporting bias, reported to be present in more than 50% of currently published RCTs^9^. An estimated 30% of RCTs are prematurely discontinued and 20% of completed trials remain unpublished 10 years after completion^10^. Inclusion bias remains a matter of concern, with less than 10% of all cancer patients being included in RCTs^3^.

Compared with RCTs, RBSs have several potential advantages. Registry-based studies usually require less financial resources, and can be completed in less time than RCTs, as data are already available in registries. Registries enable data collection from large patient cohorts and have a lower risk of selection bias than RCTs. The prospective collection of data independent of subsequent research questions and exact censoring routines also reduces the risk of recall bias. Furthermore, registry-based data often have good external validity and are representative of routine clinical practice in the general population, including elderly patients, patients with comorbidities, and patients from lower socioeconomic groups who are often excluded from RCTs^11–13^. In addition, large sample sizes retrieved from large registries allow the study of clinical questions that cannot be addressed in RCTs owing to statistical power limitations or time and financial constraints.

A potential disadvantage of publications based on single-country data is that the results may not necessarily be generally applicable. Collaborative multinational NPBRs may generate more representative and widely applicable study results with significantly increased patient numbers. The discordance in registry and datasheet design between national registries may complicate such cooperation^14^. Furthermore, the data included are predetermined and cannot be controlled by the researchers. Studies that require data not included in the registry dataset will need to complement the secondary (registry) data with the primary collection of additional data.

Several trial designs are used in RBSs, including single and comparative cohort observational studies, case–control studies, cross-sectional studies, nested case–control studies, and comparative effectiveness studies. Registry-based randomized controlled trials (rRCTs) are a pragmatic trial design in which RCTs are embedded in the data collection infrastructure of one or more registries. Registry-based randomized controlled trials integrate the strengths of RCTs with the advantages of registry-based research, allowing comparative effectiveness research in real-world settings and enabling more rapid recruitment at a significantly lower cost than conventional RCTs^15,16^. In addition, the high internal validity of RCTs and high external validity of RBSs are maintained. There seems to be no systematic difference between the effect estimates of rRCTs and conventional RCTs^17^.

#### Ethics and public disclosure of results from registry-based studies

Registry-based research should adhere to the requirements of the institutional ethics authorities where the research was conducted, and protocols should be registered in publicly accessible databases in accordance with the 2013 Declaration of Helsinki. Furthermore, studies should adhere to reporting guidelines for specific study formats, such as the Strengthening the Reporting of Observational Studies in Epidemiology (STROBE) or Strengthening the Reporting of Cohort, Cross-sectional and Case-control Studies in Surgery (STROCSS) guidelines in observational studies and the Consolidated Standards of Reporting Trials (CONSORT) guidelines in rRCTs^18,19^.

### Registry-based pancreatic cancer surgery research

**Figure 1:**
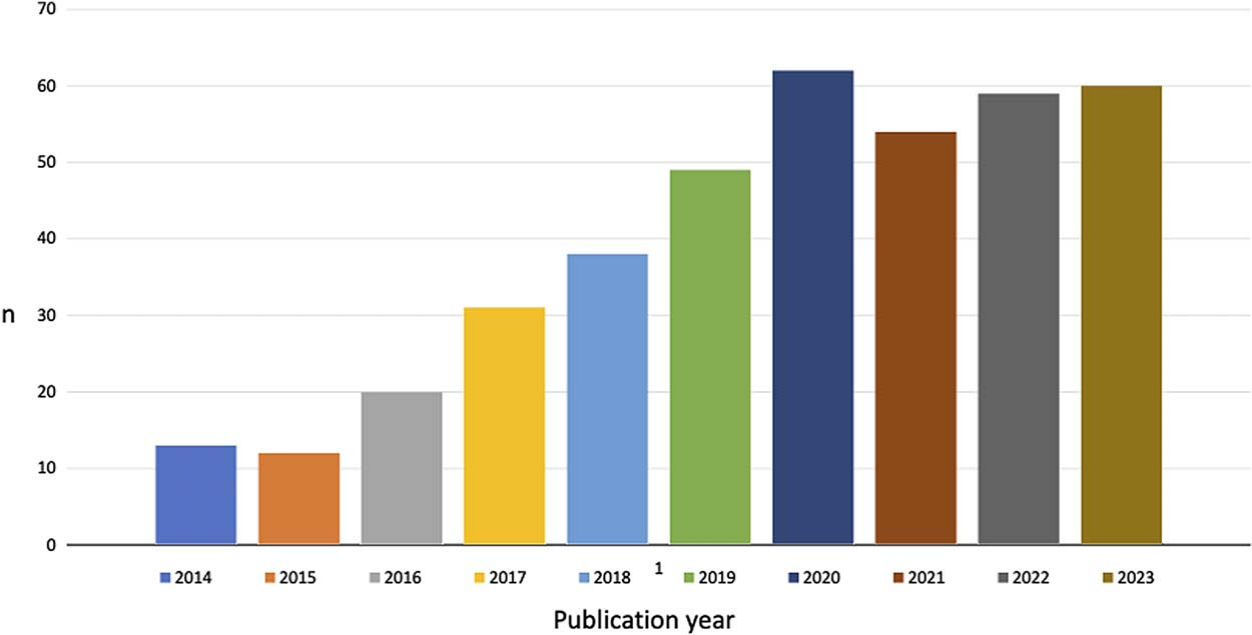
The annual number of publications from 1st January 2014 to 31st December 2023 that reported on pancreatic cancer surgery based on data retrieved from at least one national population-based pancreatic cancer surgery registry or database.

A PubMed search was performed to identify peer-reviewed original articles published between the 1st January 1995 to the 31st December 2023 that reported on any aspect of pancreatic cancer surgery based on data retrieved from at least one national population-based pancreatic cancer registry/database published in English (search strategy summarized in Supplementary Table 1, Supplemental Digital Content 1, http://links.lww.com/JS9/C325). The search yielded 1047 results, of which 441 fulfilled the inclusion criteria. Following the first paper published in 1995, 43 articles were published up to 2013^20^. The annual number of publications from 2014 to 2023 is shown in Figure 1. From 2016, a steep increase was seen, with more than 50 consistently published annually between 2020 and 2023.

A detailed analysis of the 60 articles published from 1st January to 31st December 2023 was performed (Table 1)^21–80^. A total of 15 national registries/databases were used, of which five were pancreas-specific (Table 2). Four registries, the Danish Pancreatic Cancer Database (DPCD), the Dutch Pancreatic Cancer Audit (DPCA), the Dutch Locally Advanced Pancreatic Cancer Registry (LAPCR), and Swedish National Pancreatic and Periampullary Cancer Registry (SNPPCR) are dedicated to pancreatic cancer. Only six of the publications were based on these dedicated registries/databases^21,26,34,67,70,78^. In three studies, data from two and in one study, data from four registries/databases were included^26,27,35,58^. The databases most often used as data sources were the National Cancer Database (NCDB), which was used in 32 articles (53.3%), followed by the National Surgical Quality Improvement Program (NSQIP) in nine (15%) and the Surveillance, Epidemiology, and End Results (SEER) database in six (10%). Data from USA-based registries/databases was used in 48 articles (80%), followed by the Netherlands with seven articles (7.6%) and Sweden and Germany with three (5%) each. A cross-sectional study format was used in four studies^35,46,47,54^. The remaining articles were single or comparative cohort studies. No rRCTs were performed.

**Table 1:** List of articles reporting on pancreatic cancer surgery research based on data from at least one national population-based registry/database published from 1st January to 31st December 2023

				Validation reported			
Publication	Registry	Country	Inclusion period	Coverage	Completeness	Accuracy	Guidelines used
Aaquist *et al*.^21^	DPCD	Denmark	2015–2019	Yes	Yes	Yes	STROBE
Altimari *et al*.^22^	NCDB	USA	2004–2016	Yes	No	No	No
Alwatari *et al*.^23^	NSQIP	USA	2014–2019	No	No	No	No
Amin *et al*.^24^	NCDB	USA	2006–2016	Yes	No	No	No
Anteby *et al*.^25^	NCDB	USA	2004–2017	No	No	No	No
Augustinus *et al*.^26^	NSQIP, DPCA, DGAV StuDoQ, SNPPCR	Multi	2018–2020	No	No	No	STROBE
Augustinus *et al*.^27^	NCR, PALGA	Netherlands	2009–2019	No	No	No	No
Boutros *et al*.^28^	NCDB	USA	2011–2017	Yes	No	No	No
Dalmacy *et al*.^29^	SEER	USA	2004–2015	No	No	No	No
Davis *et al*.^30^	NSQIP	USA	2014–2019	Yes	No	No	No
Ghukasyan *et al*.^31^	NCDB	USA	2004–2015	Yes	Yes	No	STROBE
Harris *et al*.^32^	NSQIP	USA	2014–2019	No	No	No	No
Hester *et al*.^33^	NCDB	USA	2006–2015	Yes	No	No	No
Holmberg *et al*.^34^	SNPPCR	Sweden	2010–2019	No	No	No	STROBE
Honselmann *et al*.^35^	DGAV StuDoQ	Germany	2013–2017	No	No	No	No
Hopstaken *et al*.^36^	NCR	Netherlands	2015–2020	No	No	No	No
Huerta *et al*.^37^	NRD	USA	2010–2014	No	No	No	No
Jung *et al*.^38^	NCDB	USA	2006–2016	No	No	No	No
Jung *et al*.^39^	HIRA	Korea	2013–2015	No	No	No	No
Kakish *et al*.^40^	NCDB	USA	2004–2020	Yes	No	No	No
Kalabin *et al*.^41^	NCDB	USA	2004–2018	Yes	No	No	STROBE
Kelly *et al*.^42^	NCDB	USA	2006–2019	No	No	No	No
Kemp Bohan *et al*.^43^	NCDB	USA	2004–2016	No	No	No	No
Kwon *et al*.^44^	SEER	USA	2000–2016	No	No	No	No
Lee *et al*.^45^	NCDB	USA	2006–2016	No	No	No	No
Li *et al*.^46^	SEER	USA	2002–2019	No	No	No	No
Li *et al*.^47^	SEER	USA	2000–2018	No	No	No	No
Lima *et al*.^48^	NCDB	USA	2011–2018	Yes	No	No	No
Lima *et al*.^49^	NCDB	USA	2006–2019	No	No	No	No
Luo *et al*.^50^	NCDB	USA	2004–2020	No	No	No	No
Maduekwa *et al*.^51^	NCDB	USA	2004–2019	Yes	No	No	No
Mangieri *et al*.^52^	NSQIP	USA	2014–2016	No	No	No	No
Mederos *et al*.^53^	NSQIP	USA	2015–2018	No	No	No	No
Nassour *et al*.^54^	NCDB	USA	2006–2019	Yes	No	No	No
Olecki *et al*.^55^	NCDB	USA	2006–2017	Yes	No	No	No
Palacio *et al*.^56^	NCDB	USA	2014–2016	No	No	No	No
Park *et al*.^57^	NCDB	USA	2006–2019	Yes	No	No	No
Petruch *et al*.^58^	NCDB, GCRG/ADT	USA/Germany	2010–2020	No	No	No	CONSORT
Powers *et al*.^59^	NCDB	USA	2004–2015	Yes	No	No	No
Quispel *et al*.^60^	PALGA	Netherlands	2014–2018	No	No	No	STROBE
Robbins *et al*.^61^	NSQIP	USA	2014–2020	No	No	No	No
Saadat *et al*.^62^	SEER	USA	2006–2015	Yes	No	No	No
Sahakayan *et al*.^63^	NORGAST	Norway	2015–2021	No	No	No	STROBE
Sahara *et al*.^64^	NSQIP	USA	2014–2020	No	No	No	No
Seldomridge *et al*.^65^	NCDB	USA	2010–2020	Yes	No	No	No
Servin Rojas *et al*.^66^	NCDB	USA	2010–2019	Yes	No	No	No
Silesen *et al*.^67^	DPCD	Danish	2011–2020	No	No	No	Yes
Spitzer *et al*.^68^	NCDB	USA	2006–2016	No	No	No	No
Stiles *et al*.^69^	NCDB	USA	2010–2015	Yes	No	No	No
Stoop *et al*.^70^	DPCA/LAPC	Netherlands	2014–2020	No	No	No	STROBE
Sugawara *et al*.^71^	NCDB	USA	2010–2018	Yes	No	No	STROBE
Sugawara *et al*.^72^	NCDB	USA	2010–2017	Yes	No	No	STROBE
Valdera *et al*.^73^	NCDB	USA	2006–2017	No	No	No	No
Vawter *et al*.^74^	NSQIP	USA	2014–2019	No	No	No	No
Villano *et al*.^75^	NCDB	USA	2010–2017	Yes	No	No	STROBE
Yoo *et al*.^76^	KNHISD	Korea	2002–2019	No	No	No	No
Yun *et al*.^77^	NCDB	USA	2010–2016	No	No	No	No
Zdanowski *et al*.^78^	SNPPCR	Sweden	2010–2018	Yes	No	No	No
Ziogas *et al*.^79^	NCDB	USA	2004–2016	Yes	No	No	No
Zou *et al*.^80^	SEER	USA	2010–2019	No	No	No	No

DGAV, StuDoQ German pancreatic surgery registry; DPCA, Dutch Pancreatic Cancer Audit; DPCD, Danish Pancreatic Cancer Database; DPCD, Danish Pancreatic Cancer Database; GCRG/ADT, German Cancer Registry Group of the Society of German Tumor Centers; HIRA, Korean Health Insurance Review and Assessment Service; KNHISD, Korean National Health Insurance Service database; LAPC, Locally Advanced Pancreatic Cancer Registry; NCDP, National Cancer Database; NCR, Netherlands Cancer Registry; NORGAST, Norwegian Registry for Gastrointestinal Surgery; NRD, Nationwide Readmissions Database; NSQIP, National Surgical Quality Improvement Program; PALGA, Dutch Nationwide Pathology Database/Dutch Pathology Registry; SEER, National Cancer Surveillance Epidemiology and End Results Program; SNPPCR, Swedish National Pancreatic and Periampullary Cancer Registry.

**Table 2:** The registries/databases that were sources of data in publications on pancreatic cancer surgery published in 2023 based on data retrieved from at least one national population-based registry/database.

Country	Registry	Articles (*n*)
Denmark	Danish Pancreatic Cancer Database (DPCD)	2
Germany	German Cancer Registry Group of the Society of German Tumor Centers (GCRG/ADT)	1
	German Pancreatic Surgery Registry (DGAV StuDoQ)	2
Norway	Norwegian Registry for Gastrointestinal Surgery (NORGAST)	1
Netherlands	Netherlands Cancer Registry (NCR)	2
	Dutch Pancreatic Cancer Audit (DPCA)	2
	Locally advanced Pancreatic cancer registry (LAPCR)	1
	Dutch Nationwide Pathology Database / Dutch Pathology Registry (PALGA)	2
South Korea	Korean National Health Insurance Service Database (KNHIHD)	1
	Korean Health Insurance Review and Assessment Service (HIRA)	1
Sweden	Swedish National Pancreatic and Periampullary Cancer Registry (SNPPCR)	3
United States	National Cancer Database (NCDB)	32
	National Cancer Surveillance Epidemiology and End Results Program (SEER)	6
	National Surgical Quality Improvement Program (NSQIP)	9
	Nationwide Readmissions Database (NRD)	1

The use of the STROBE guidelines was reported in 12 articles^21,26,31,34,41,60,63,67,70–72,75^. In one article, a cross-validation study of two national registries, the CONSORT guidelines, designed for RCTs were used^58^.

Validation in terms of coverage was mentioned in 23 articles (38.3%). In 21 articles based on NCDB, data completeness was mentioned, all referring to a coverage of around 70% (approximately 70%, more than 70%, about 70%, over than two-thirds). Most referenced the reported coverage with references to previously published articles, and some referenced the homepage of the respective registries^5,81,82^. In one article, based on data from the SNPPCR, a referenced coverage of >93% was mentioned, and in one SEER-based paper, a referenced coverage of 48%^62,78^. The completeness of data was quantified in one single paper. In a study based on NCDB data by Ghukasyan *et al*.^31^, the percentage of incomplete cases ranged between 43.5 and 56.5%. Accuracy of data was mentioned but not quantified in one paper^21^. The presence of missing data was acknowledged and at least partially shown in 13 papers^21,25,34,45,46,48,49,63,66,70,74,75,78^. In 17 papers, missing data of at least some of the key parameters were regarded as exclusion criteria^23,27,31,36,41,43,47,53,55,59,61,65,71–73,76,80^. For studies based on USA registries, inclusion periods ranged from 2000 to 2020, whereas the inclusion periods for European studies ranged from 2009 to 2020.

A wide variety of topics was addressed in the articles. Notably, in nine articles, the impact of race on treatment and outcomes was reported, all based on USA registries/databases^23–25,29,33,39,48,51,55^. Six of the articles were based on NCDB data, two on NSQIP data, and one on SEER data. Aspects of chemotherapy were addressed in nine articles^23,36,54,57,70–73,80^. Six of the articles were based on NCDB data, two on Dutch registries, and one on SEER data.

## Discussion

The increase in the number of publications on pancreatic cancer surgery based on national population-based registries or databases over, in particular, the last decade is encouraging and has without doubt contributed to the body of knowledge on many aspects of pancreatic surgery. However, a reflection on the quality of the registries/databases from which the data for articles is obtained is warranted, as is a critical assessment of articles originating from these registries.

Most publications still originate from comprehensive registries/databases that are not specifically designed for pancreatic cancer. The data entered into these registries/databases are generic and not optimized for capturing information crucial to pancreatic cancer-related research specifically. In the detailed analysis of articles published in 2023, only 10% of publications were based on pancreatic cancer registries/databases. Notably, almost 80% of publications were completely or partially based on data from three USA registries/databases. In two of these publications, patient data from 2000, and in another 11, data from 2004 were included^22,25,29,31,40,41,43,44,47,50,51,59,79^. The expiry date of registry-based data relevant to pancreatic cancer surgery depends, amongst other factors, on the outcomes that are assessed, such as prognostic-related outcomes and treatment outcomes. Whereas historical data may well be appropriate for assessing some prognostic-related outcomes, the advancements in treatments of pancreatic cancers make the inclusion of almost 24-year-old data questionable.

A further concern is the lack of use or inappropriate use of study format-specific guidelines. The STROBE guidelines were appropriately used in only 20% of articles, and in one, the CONSORT guidelines were inappropriately used^21,26,31,34,41,58,60,63,67,70–72,75^.

The lack of reporting on the validation of the registries/databases used was disappointing. The most reported validation parameter was coverage, which was reported in 23 publications. The reporting was often imprecise, as was evident in the NCDB data-based articles where terms such as approximately, more than, and about 70% were used. Furthermore, the references used to support the completeness numbers date back as far as 2008^82^. In the limited available data from the registries and databases that were sourced data for the 2023 publications, coverage varied from as low as 10 to 100%. The DGAV StuDoQ registry reported an estimated coverage of between 10 and 20% for pancreatic resections, whereas the GCRG/ADT has reported a coverage of 100%^83,84^. Data completeness was reported in only two articles and accuracy in one^21,31^. A further concern was that information on missing data and how missing data was dealt with was reported in less than 25% of publications.

Finally, the ready availability of registry/database data carries an inherent risk of publishing multiple articles on the same data with overlapping or similar endpoints. For example, in the 2023 publications, there were nine articles based on USA registries/databases that reported on the impact of race on treatment and outcomes^23–25,29,33,39,48,51,55^. Notably, six of the articles were based on NCDB data and included assessments of patients from identical or vastly overlapping inclusion periods.

## Conclusion and future perspectives

The important role that national population-based registry research plays in pancreatic cancer surgery research is indisputable. There are, however, several concerning shortcomings that will need to be addressed. The establishment of dedicated pancreatic cancer registries/databases that include data specific to the disease needs to be encouraged. This could be facilitated by guidelines for the design of pancreatic cancer, in particular optimizing registries so that data allow rRCT, a potentially valuable but under-utilized trial design in pancreatic cancer research^85^. In particular, the inclusion of key variables reporting on different pancreatic surgery-related topics as well as different registry/database-based study formats should be included. Standardization of core parameters that should be included in pancreatic cancer registries will promote and facilitate multinational registry-based research, which is currently used to a very limited extent in pancreatic cancer research. In a study in which four nationwide or multi-center pancreatic surgery registries from the United States, Germany, the Netherlands, and Sweden were compared, it was found that 20 of 55 (36.4%) core parameters were not available in one or more of the registries^14^.

The inappropriate use of historical data which is prevalent in current research, can be prevented with the guidance of appropriate inclusion periods depending on study topics, study design, and outcomes. The use of appropriate study format-specific guidelines should be encouraged or even be made mandatory for publication. Guidelines reporting validation parameters (coverage, completeness, and accuracy) should be established, and inclusion of the measures or lack thereof should be mandatory in publications. Reporting missing data should be mandatory. There should also be awareness of the risks of double publication, especially from databases where oversight from the registry/database administration on published articles is lacking^28^.

Finally, dedicated funding for financing the establishment of new and maintenance and improvement of existing registries is essential to improve the quality of the registries and, by inference, also publications based on registry data. Such improvements will further entrench the important role that registry-based have in generating unique patient-centered evidence for optimizing patient management and outcomes.

## Ethical approval

This invited article is purely based on already published research, and no patient data or outcomes have been reported. It did not require ethical approval from our institutional ethics committee.

## Consent

This invited article is purely based on already published research, and no patient data or outcomes have been reported. Informed written consent was, therefore, not relevant.

## Sources of funding

The research that was performed for this Invited Special Paper did not receive any funding.

## Author contribution

Professor Eduard Jonas, the primary author of the Invited Special Paper, was responsible for the study concept and design, data collection, data analysis and interpretation, and wrote the first draft of the paper.

Professor Christo Kloppers reviewed the data collected and the results included in the manuscript and performed a critical review of the final manuscript.

## Conflicts of interest disclosure

Neither of the authors has any conflicts of interest to report.

## Guarantor

Professor Eduard Jonas.

## Data availability statement

Datasets regarding the literature search in the manuscript have been included in Supplementary Table 1. The datasets extracted from the review of the search results are available upon request.

## Provenance and peer review

This is an invited paper.

## Supplementary Material

SUPPLEMENTARY MATERIAL
