# Supplementary material for: The role of national population-based registries in pancreatic cancer surgery research
Source: Int J Surg. 2024 Apr 4;110(10):6155–62. doi: 10.1097/JS9.0000000000001405 (PMC11487038; doi:10.1097/JS9.0000000000001405)
Supplement: SUPPLEMENTARY MATERIAL [file js9-110-6155-s001.docx]

Table S1. PubMed (Medline) search strategy

‘

| # | **Category** | **Search Term** |
| --- | --- | --- |
| #1 | Key word | pancreas |
| #2 | Key word | pancreatic |
| #3 |  | #1 OR #2 OR |
| #4 | Key word | cancer |
| #5 | Key word | cancers |
| #6 | Key word | carcinoma |
| #7 | Key word | carcinomas |
| #8 | Key word | adenocarcinoma |
| #9 | Key word | adenocarcinomas |
| #10 | Key word | tumour |
| #11 | Key word | tumours |
| #12 |  | #4 OR #5 OR #6 OR #7 OR #8 OR #9 OR #10 OR #11 |
| #13 | Key word | resection |
| #14 | Key word | resections |
| #15 | Key word | pancreatectomy |
| #16 | Key word | pancreatectomies |
| #17 | Key word | pancreatoduodenectomy |
| #18 | Key word | pancreato-duodenectomy |
| #19 | Key word | pancreaticoduodenectomy |
| #20 | Key word | pancreatico-duodenectomy |
| #21 | Key word | Whipple |
| #22 |  | #13 OR #14 OR #15 OR #16 OR #17 OR #18 OR #19 OR #20 OR #21 |
| #23 | Key word | national |
| #24 | Key word | nationwide |
| #25 | Key word | nation-wide |
| #26 | Key word | country-wide |
| #27 | Key word | countrywide |
| #28 |  | #23 OR #24 OR #25 OR #26 OR “27 |
| #29 | Key word | registry |
| #30 | Key word | registries |
| #31 | Key word | database |
| #33 | Key word | databases |
| #34 | Key word | register |
| #35 | Key word | registers |
| #36 | Key word | repository |
| #37 | Key word | repositories |
| #38 |  | #29 OR #30 OR #31 OR #32 OR #33 OR #34 OR #35 OR #36 OR #37 |
| #39 |  | #3 AND #12 AND #28 AND #37  Limit (date): 01/01/1995 – 31-12-2023  Limit (language): English |
